# Green Tea Consumption Affects Cognitive Dysfunction in the Elderly: A Pilot Study

**DOI:** 10.3390/nu6104032

**Published:** 2014-09-29

**Authors:** Kazuki Ide, Hiroshi Yamada, Norikata Takuma, Mijong Park, Noriko Wakamiya, Junpei Nakase, Yuuichi Ukawa, Yuko M. Sagesaka

**Affiliations:** 1 Department of Drug Evaluation and Informatics, Graduate School of Pharmaceutical Sciences, University of Shizuoka, 52-1 Yada, Suruga-ku, Shizuoka 422-8526, Japan; E-Mails: s13403@u-shizuoka-ken.ac.jp (K.I.); parkmjjp@yahoo.co.jp (M.P.); m08129@ u-shizuoka-ken.ac.jp (N.W.); 2 White Cross Nursing Home, 2-26-1 Suwa-cho, Higashimurayama, Tokyo 189-0021, Japan; E-Mail: hide1844@wa2.so-net.ne.jp; 3 Central Research Institute, ITO EN, Ltd., 21 Mekami, Makinohara, Shizuoka 421-0516, Japan; E-Mails: j-nakase@itoen.co.jp (J.N.); y-ukawa@itoen.co.jp (Y.U.); y-sagesaka@itoen.co.jp (Y.M.S.)

**Keywords:** green tea, oral administration, cognitive function, elderly

## Abstract

Green tea is known to have various health benefits for humans. However, the effect of green tea consumption on cognitive dysfunction remains to be clinically verified. We conducted a clinical study to investigate the effects of green tea consumption on cognitive dysfunction. Twelve elderly nursing home residents with cognitive dysfunction (Mini-Mental State Examination Japanese version (MMSE-J) score: <28) participated in the study (2 men, 10 women; mean age, 88 years). The participants consumed green tea powder 2 g/day for 3 months. After three months of green tea consumption, the participants’ MMSE-J scores were significantly improved (before, 15.3 ± 7.7; after, 17.0 ± 8.2; *p* = 0.03). This result suggests that green tea consumption may be effective in improving cognitive function or reducing the progression of cognitive dysfunction; however, long-term large-scale controlled studies are needed to further clarify the effect.

## 1. Introduction

In rapidly aging societies around the world, the number of patients with cognitive dysfunction, particularly dementia, is gradually increasing [1]. Dementia affects 5.4% of people over 65 years of age worldwide, and its prevalence increases with age [2]. There are several pharmaceutical and non-pharmaceutical treatments for dementia; however, thus far, no fundamental curative therapy has been established [3,4].

Green tea, one of the commonly consumed beverages in Asian countries, is known to have various health benefits [5,6,7]. A number of experimental studies *in vitro* and *in vivo* have shown the neuroprotective effects of green tea and its components, such as catechins and theanine [8,9,10,11]. Anti-oxidative and anti-inflammatory effects of these components have been reported [10,11,12], and such effects may contribute to neuroprotection. Regarding cognitive function, catechins have been reported to improve performance on cognition tests in rodent models of dementia, such as the Morris water maze, probe test, and passive avoidance test [10]. In mice, theanine has also been shown to attenuate memory impairment induced by amyloid protein, an Alzheimer’s disease trigger protein [9].

Furthermore, several human epidemiological studies have shown a relationship between tea consumption and cognitive function [13,14,15,16,17,18]. One study showed a negative association between green tea consumption and the prevalence of cognitive impairment in elderly individuals over 70 years old [13]. Similar negative association has also been reported in other observational studies on tea consumption [14,15,16,18]. These studies suggest that tea consumption effects cognitive function; however, only limited interventional studies have been reported, and these effects remain to be clinically verified [19,20]. One interventional study used a supplement called LGNC-07 containing 1440 mg green tea extract and 240 mg theanine as a daily dose [19]. It should be noted that LGNC-07 is a green-tea-based supplement, not green tea as it is typically drunk, thus, it is important to also study the effect of ordinary daily green tea consumption patterns on cognitive function.

In addition to the effect of green tea consumption on cognitive function, several studies have shown that it also reduces the risk of developing hypertension [21] and lowers both total cholesterol (TC) concentration and low density lipoprotein cholesterol (LDL-C) concentration in adults [22]. Clinical significance of these effects is still inconclusive; however, hypertension and dyslipidemia are risk factors for atherosclerosis [23,24], and atherosclerosis is in turn related to cognitive dysfunction. Therefore, green tea consumption may also reduce the progression of cognitive dysfunction indirectly by reducing the effect of these related health problems on atherosclerosis.

Based on this previous research, we conducted a clinical study to investigate the effects of green tea consumption on cognitive dysfunction and atherosclerotic risk factors in the elderly.

## 2. Experimental Section

### 2.1. Subjects

This study was conducted from July to September 2012 at the White Cross Nursing Home in Higashi-Murayama, Japan. Recruitment was performed at the nursing home by posters. Fifteen elderly residents with cognitive impairment were enrolled. Inclusion criteria were as follows: (1) >65 years of age; (2) ability to orally ingest green tea powder; (3) no consumption of supplements with antioxidant effects (vitamins E, C, and A, and β-carotene) during the study period; and (4) a Mini-Mental State Examination-Japanese version (MMSE-J) score of <28 [25]. Exclusion criteria were: (1) tea allergy; (2) severe cardiac, respiratory, hepatic, or renal dysfunction; and (3) severe anemia. The diagnoses of the patients were simply taken from the medical records at White Cross Hospital in Higashi-Murayama, Japan.

Written informed consent was obtained from both the subjects and their caregivers prior to enrollment. The study protocol was approved by the Ethics Committee of the University of Shizuoka (No. 23-27, approved on 11 May, 2012) and conducted in accordance with the Declaration of Helsinki. This pilot study was registered with Clinical Trials.gov (NCT 01594086).

### 2.2. Study Design

The following baseline characteristics of subjects were recorded: age, sex, underlying diseases, complications, medication, alcohol consumption, smoking habits, tea or supplement consumption habits, activity of daily living, and brain magnetic resonance imaging (MRI) or computed tomography (CT) findings.

The subjects were asked to consume green tea powder (2 g/day, containing 227 mg catechins and 42 mg theanine, manufactured by ITO EN Ltd. (Tokyo, Japan)) during meals for a period of 3 months. The consumption of other supplements that could have antioxidant effects was prohibited during the intervention period and for a seven-day washout period prior to the start of the intervention. Subjects were advised to maintain their customary intake of home-brewed green tea or tea beverages during the study period. The caregiving staff at the nursing home kept a diary for each subject in which they recorded the daily intake of green tea powder, the amount of home-brewed green tea or tea beverages consumed each day, any changes in the health of subjects or in the administration of medication, and the occurrence of any adverse events.

MMSE-J tests were performed to assess the cognitive function of subjects. In addition, the following data was collected: blood pressure; serum lipid levels, including TC, LDL-C, HDL-C, and triglycerides; and blood glucose levels. All tests were performed at baseline and again after three months of green tea consumption.

### 2.3. Statistical Analysis

Changes in MMSE-J scores, including scores for specific cognitive domains, as well as clinical and laboratory values obtained at baseline and three months after the start of green tea consumption were determined by paired *t*-test or Wilcoxon signed-rank test. Statistical significance was set at *p* < 0.05. All statistical procedures were performed with IBM SPSS version 20.0 for Windows (IBM Corp., Armonk, NY, US).

## 3. Results

A total of 15 nursing home elderly residents and their caregivers gave written informed consent, and were assessed for eligibility. One resident was excluded according to the exclusion criteria. Two residents were excluded from the study after it had begun due to the retracting of their consent, so it was not possible to obtain intervention data for these individuals. A total of 12 subjects (2 men, 10 women) completed the study. During the study period, a subject was hospitalized due to a hip fracture but resumed participation 36 days after the initial enrollment. The mean age of subjects was 88 ± 7.6 (range, 70–98) years; eight subjects had vascular dementia, three had Alzheimer’s disease and one had dementia with Lewy body. The characteristics of subjects are reported in Table 1. The MMSE-J score distribution of the subjects prior to the intervention was as follows: 24–27 (mild cognitive impairment (MCI)), two subjects (16.7%); 10–23 (mild and moderate dementia), six subjects (50.0%); and 0–9 (severe dementia), four subjects (33.3%). Adherence to the green tea powder consumption protocol was 99.7%.

**Table 1:** Clinical characteristics of study subjects.

Clinical Characteristic	
Number of subjects	12
Age, mean ± SD (range)	88 ± 7.6 (70–98)
*Sex, n (%)*	
Men	2 (16.7)
Women	10 (83.3)
*Underlying disease, n (%)*	
Alzheimer’s disease	3 (25.0)
Vascular dementia	8 (66.7)
Dementia with Lewy bodies	1 (8.3)
*MMSE-J score, n (%)*	
24–27 (MCI)	2 (16.7)
10–23 (mild to moderate)	6 (50.0)
0–9 (severe)	4 (33.3)
*Complication ^a^, n (%)*	
Hypertension	8 (66.7)
Diabetes	2 (16.7)
Hyperuricemia	1 (8.3)
*Concomitant drug ^a^, n (%)*	
Antihypertensive drug	8 (66.7)
Drug for hyperuricemia	2 (16.7)
Antidiabetic drug	1 (8.3)
Drug for dementia	1 (8.3)
*Activities of daily living*	
Independence	0 (0.0)
Some assistance is necessary	12 (100)
*Usual tea consumption*	
Green tea, *n* (%)	12 (100)
Mean ± SD, mL/day	680 ± 229.8
Others ^b^, *n* (%)	8 (66.7)
Mean ± SD, mL/day	85 ± 63.7
Alcohol use, *n* (%)	0 (0.0)
Smoking, *n* (%)	2 (16.7)
Dietary supplements, *n* (%)	0 (0.0)

MMSE-J, Mini-Mental State Examination Japanese version; MCI, Mild cognitive impairment. ^a^ More than one choice was possible, ^b^ black tea or oolong tea.

Changes in MMSE-J scores before and after the intervention are shown in Table 2. Total MMSE-J scores (mean ± SD) taken at baseline were significantly improved after three months of green tea consumption (before, 15.3 ± 7.7; after, 17.0 ± 8.2; *p* = 0.03). In terms of specific cognitive domains, the baseline scores for short-term memory (registration and recall) were significantly improved after the intervention (before, 2.0 ± 1.8; after, 3.2 ± 1.8; *p* = 0.01) (Table 2).

In *post hoc* analysis for vascular dementia (*n* = 8), the MMSE-J scores (mean ± SD) were significantly improved after the intervention (before, 18.4 ± 6.5; after, 20.6 ± 6.7; *p* = 0.03), and short-term memory were also improved significantly (before, 2.6 ± 1.6; after, 4.0 ± 1.2; *p* = 0.04) (Table 2).

**Table 2:** MMSE-J scores before and after 3 months of green tea consumption.

Cognitive Function (MMSE-J Score)	Green Tea Consumption (2 g/day)		
	Before	After	*p* Value
*All subjects (n = 12)*			
Total MMSE-J score (max, 30)	15.3 ± 7.7	17.0 ± 8.2	0.03 ^t^
Orientation (max, 10)	4.2 ± 3.1	4.3 ± 3.9	0.96 ^w^
Short-term memory (max, 6)	2.0 ± 1.8	3.2 ± 1.8	0.01 ^t^
Attention and calculation (max, 5)	2.1 ± 2.0	2.0 ± 2.3	0.91 ^w^
Language (max, 8)	6.7 ± 1.7	6.9 ± 1.4	0.46 ^w^
Visual construction (max, 1)	0.4 ± 0.5	0.7 ± 0.5	0.08 ^w^
*Vascular dementia (n = 8)*			
Total MMSE-J score (max, 30)	18.4 ± 6.5	20.6 ± 6.7	0.03 ^t^
Orientation (max, 10)	5.1 ± 3.3	5.8 ± 3.8	0.11 ^t^
Short-term memory (max, 6)	2.6 ± 1.6	4.0 ± 1.2	0.04 ^w^
Attention and calculation (max, 5)	2.8 ± 1.9	2.5 ± 2.3	0.68 ^w^
Language (max, 8)	7.4 ± 0.9	7.5 ± 0.9	0.32 ^w^
Visual construction (max, 1)	0.5 ± 0.5	0.8 ± 0.5	0.16 ^w^
*Stratified analysis at each stages of cognitive dysfunction*			
Total MMSE-J score (max 30)			
MCI (*n* = 2)	26.5 ± 0.7	29.0 ± 1.4	0.34 ^t^
Mild to moderate (*n* = 6)	17.3 ± 3.7	18.8 ± 4.3	0.19 ^t^
Severe (*n* = 4)	6.8 ± 1.7	8.3 ± 3.4	0.32 ^t^

Values: Mean ± SD. Each *p* value was calculated using the following statistical method: ^t^ paired *t*-test, ^w^ Wilcoxon signed-rank test. MMSE-J, Mini-Mental State Examination Japanese version; MCI, Mild cognitive impairment.

After three months of green tea consumption, the triglyceride (TG) levels of subjects were significantly lower than those measured at baseline (124 ± 80 mg/dL *vs* 103 ± 57 mg/dL; *p* = 0.04). However, blood pressure, lipid profiles (TC, LDL-C, and high density lipoprotein cholesterol (HDL-C)), and blood glucose levels were not significantly different (Table 3).

No serious adverse events associated with green tea consumption were observed during the study period.

**Table 3:** Values of atherosclerotic factors before and after 3 months of green tea consumption.

Atherosclerotic factor	Green Tea Consumption (2 g/day)		
	Before	After	*p* Value
*All subjects (n = 12)*			
Blood pressure			
SBP (mmHg)	119 ± 19	126 ± 19	0.32 ^t^
DBP (mmHg)	65 ± 13	70 ± 12	0.19 ^t^
Serum lipid levels			
TC (mg/dL)	190 ± 33	189 ± 28	0.84 ^t^
HDL-C (mg/dL)	47 ± 18	48 ± 16	0.78 ^t^
LDL-C (mg/dL)	112 ± 24	112 ± 27	0.97 ^t^
TG (mg/dL)	124 ± 80	103 ± 57	0.04 ^w^
Blood glucose levels			
FPG (mg/dL)	124 ± 52	124 ± 38	0.86 ^w^
HbA1c (%)	5.3 ± 0.6	5.2 ± 0.6	0.14 ^w^

Values: Mean±SD. Each *p* value was calculated using the following statistical method: ^t^ paired *t*-test, ^w^ Wilcoxon signed-rank test. SBP, Systolic blood pressure; DBP, Diastolic blood pressure; TC, total cholesterol; HDL-C, high density lipoprotein cholesterol; LDL-C, low density lipoprotein cholesterol; TG, Triglyceride; FPG, fasting plasma glucose; HbA1c: hemoglobin A1c.

## 4. Discussion

In this pilot study conducted to investigate the effect of green tea consumption on cognitive dysfunction and atherosclerotic risk factors in the elderly, we found that three months of green tea consumption improved cognitive dysfunction based on MMSE-J score changes. Among MMSE-J domains, the short-term memory domain was especially improved. These results support the findings of previous epidemiological studies, and additionally demonstrate that green tea improves cognitive function or reduces the progression of cognitive dysfunction even at the relatively low catechin and theanine concentrations that can be obtained from ordinary levels of daily green tea intake. The green tea powder used as a daily dose in this study contained as its main bioactive components 227 mg of catechins and 42 mg of theanine, concentrations that are approximately equal to two to four cups of bottled or home-brewed green tea.

The effect on cognitive function estimated by MMSE-J in this study may be partially explained in terms of basic studies on the bioactive components of green tea, such as catechins and theanine. The anti-oxidative and anti-inflammatory properties of these components [10,11,12] may have contributed to the effect of green tea consumption on cognitive function. In addition to these properties, recent findings have suggested that green tea may exert its neuroprotective effect through a variety of different mechanisms, including: tea polyphenols inhibiting acetylcholinesterase, which is a target for Alzheimer’s disease medications [26]; green tea extract regulating the secretion of stress hormones such as corticosterone, which is related to cognitive function [27]; and l-theanine modulating serotoninergic [28,29,30], dopaminergic [30], and GABAergic [31] neurotransmission in brain. In particular, research on acetylcholinesterase inhibition has shown that tea polyphenols, including catechins and theanine, also blunted scopolamine-induced learning and memory impairment in model mice [26]. In addition, other ingredients, such as caffeine, might also be related to the improvement of MMSE-J scores by alerting influence [32]. In human, neural modulation related to cognitive function by green tea consumption have been largely uncertain. However, recently, an enhancement of parieto-frontal connectivity by green tea consumption was reported [33]. Parieto-frontal connectivity contributes working memory processing; therefore, it might be related in part to the effect of green tea on the improvement of MMSE-J score.

*Post hoc* analysis of vascular dementia data (*n* = 8) showed that total MMSE-J scores and short-term memory domain scores were significantly improved after three months of green tea consumption. Vascular dementia, the second most common type of dementia after Alzheimer’s disease, is characterized by cognitive deficit of cerebrovascular origin. Our results indicate that green tea has potential as a neuroprotective agent, especially for vascular dementia.

Individual MMSE-J scores tended to improve slightly regardless of the severity of cognitive dysfunction; however, a significant difference was not observed in the stratified analysis at each stage of cognitive dysfunction. One reason for this may be the small number of participants in this study.

There are several limitations in this study. Non-blinded, non-placebo controlled design is the main limitation. First, the participants believed that they were taking a compound that might help them, and it may induce a placebo effect on the MMSE-J scores. Second, the MMSE-J were administered twice at the three-month interval. A test-retest effect on the examination could not be eliminated.

In addition, the participants of this study were regular green tea drinker. The nursing home care and diet was not changed during study period, but baseline green tea drinking elevates the catechin and theanine consumptions, and it might affect the changes of MMSE-J scores and atherosclerotic risk factors.

Not measuring depression and other neuropsychiatric symptoms are also possible limitations. Association between late life depression and dementia has been reported [33,34,35,36]; therefore, it is not ruled out whether cognitive impairment is secondary to the improvement on mood. In future studies, study designs should be improved; a blinded, placebo controlled design is adequate to evaluate the efficacy of green tea consumption.

Our findings related to atherosclerotic risk factors showed that serum TG levels were significantly lowered. This suggests that green tea may be protective against vascular atherosclerosis in the elderly. The TG-lowering effect of green tea is also supported by the previously-reported meta-analysis by Zheng *et al.* [22]. However, clinical significance of the TG-lowering effects and its relationship with cognitive function were still inconclusive, and the other atherosclerotic risk factors assessed in this study, including LDL-C and HDL-C, did not show a significant change. The fact that the inclusion and exclusion criteria of this study were not focused on patients with atherosclerotic risk factors, and the short term intervention of this study are limitations; future studies on patients with atherosclerotic risk factors with long study period should more clearly reveal both the effect of green tea consumption on these risk factors and the relationship between the risk factors and cognitive function.

## 5. Conclusions

In conclusion, our results suggest that green tea consumption may be effective in improving cognitive function or reducing the progression of cognitive dysfunction in elderly individuals, and that it may similarly reduce the progression of vascular dementia. However, there are several limitations related to the study design. Additional long-term large-scale randomized controlled studies are needed both to establish evidence for the effect of green tea consumption on cognitive dysfunction, and to reveal the relationship between this effect and atherosclerotic risk factors.

## References

[1] Prince M., Bryce R., Albanese E., Wimo A., Ribeiro W., Ferri C.P. (2013). The global prevalence of dementia: A systematic review and metaanalysis. Alzheimer’s Dement..

[2] Sorbi S., Hort J., Erkinjuntti T., Fladby T., Gainotti G., Gurvit H., Nacmias B., Pasquier F., Popescu B.O., Rektorova I. (2012). EFNS-ENS Guidelines on the diagnosis and management of disorders associated with dementia. Eur. J. Neurol..

[3] Huang Y., Mucke L. (2012). Alzheimer mechanisms and therapeutic strategies. Cell.

[4] Citron M. (2010). Alzheimer’s disease: Strategies for disease modification. Nat. Rev. Drug Discov..

[5] Johnson R., Bryant S., Huntley A.L. (2012). Green tea and green tea catechin extracts: An overview of the clinical evidence. Maturitas.

[6] Chacko S.M., Thambi P.T., Kuttan R., Nishigaki I. (2010). Beneficial effects of green tea: A literature review. Chin. Med..

[7] Cabrera C., Artacho R., Gimenez R. (2006). Beneficial effects of green tea—A review. J. Am. Coll. Nutr..

[8] Lee J.W., Lee Y.K., Ban J.O., Ha T.Y., Yun Y.P., Han S.B., Oh K.W., Hong J.T. (2009). Green tea (−)-epigallocatechin-3-gallate inhibits beta-amyloid-induced cognitive dysfunction through modification of secretase activity via inhibition of ERK and NF-kappaB pathways in mice. J. Nutr..

[9] Kim T., Lee Y.K., Park S.G., Choi I.S., Ban J.O., Park H.K., Nam S.Y., Yun Y.W., Han S.B., Oh K.W. (2009). l-Theanine, an amino acid in green tea, attenuates beta-amyloid-induced cognitive dysfunction and neurotoxicity: Reduction in oxidative damage and inactivation of ERK/p38 kinase and NF-kappaB pathways. Free Radic. Biol. Med..

[10] Lee Y.J., Choi D.Y., Yun Y.P., Han S.B., Oh K.W., Hong J.T. (2013). Epigallocatechin-3-gallate prevents systemic inflammation-induced memory deficiency and amyloidogenesis via its anti-neuroinflammatory properties. J. Nutr. Biochem..

[11] Biasibetti R., Tramontina A.C., Costa A.P., Dutra M.F., Quincozes-Santos A., Nardin P., Bernardi C.L., Wartchow K.M., Lunardi P.S., Gonçalves C.A. (2013). Green tea (−)epigallocatechin-3-gallate reverses oxidative stress and reduces acetylcholinesterase activity in a streptozotocin-induced model of dementia. Behav. Brain Res..

[12] Coimbra S., Castro E., Rocha-Pereira P., Rebelo I., Rocha S., Santos-Silva A. (2006). The effect of green tea in oxidative stress. Clin. Nutr..

[13] Kuriyama S., Hozawa A., Ohmori K., Shimazu T., Matsui T., Ebihara S., Awata S., Nagatomi R., Arai H., Tsuji I. (2006). Green tea consumption and cognitive function: A cross-sectional study from the Tsurugaya Project 1. Am. J. Clin. Nutr..

[14] Nurk E., Refsum H., Drevon C.A., Tell G.S., Nygaard H.A., Engedal K., Smith A.D. (2009). Intake of flavonoid-rich wine, tea, and chocolate by elderly men and women is associated with better cognitive test performance. J. Nutr..

[15] Ng T.P., Feng L., Niti M., Kua E.H., Yap K.B. (2008). Tea consumption and cognitive impairment and decline in older Chinese adults. Am. J. Clin. Nutr..

[16] Arab L., Khan F., Lam H. (2013). Epidemiologic evidence of a relationship between tea, coffee, or caffeine consumption and cognitive decline. Adv. Nutr..

[17] Feng L., Li J., Ng T.P., Lee T.S., Kua E.H., Zeng Y. (2012). Tea drinking and cognitive function in oldest-old Chinese. J. Nutr. Health Aging.

[18] Feng L., Gwee X., Kua E.H., Ng T.P. (2010). Cognitive function and tea consumption in community dwelling older Chinese in Singapore. J. Nutr. Health Aging.

[19] Park S.K., Jung I.C., Lee W.K., Lee Y.S., Park H.K., Go H.J., Kim K., Lim N.K., Hong J.T., Ly S.Y. (2011). A combination of green tea extract and l-theanine improves memory and attention in subjects with mild cognitive impairment: A double-blind placebo-controlled study. J. Med. Food.

[20] Kakuda T. (2011). Neuroprotective effects of theanine and its preventive effects on cognitive dysfunction. Pharmacol. Res..

[21] Yang Y.C., Lu F.H., Wu J.S., Wu C.H., Chang C.J. (2004). The protective effect of habitual tea consumption on hypertension. Arch. Intern. Med..

[22] Zheng X.X., Xu Y.L., Li S.H., Liu X.X., Hui R., Huang X.H. (2011). Green tea intake lowers fasting serum total and LDL cholesterol in adults: A meta-analysis of 14 randomized controlled trials. Am. J. Clin. Nutr..

[23] Thies W., Bleiler L., Alzheimer’s Association (2013). 2013 Alzheimer’s disease facts and figures. Alzheimer’s Dement..

[24] Etgen T., Sander D., Bickel H., Forstl H. (2011). Mild cognitive impairment and dementia: The importance of modifiable risk factors. Dtsch. Arztebl. Int..

[25] Reisberg B., Jamil I.A., Khan S., Monteiro I., Torossian C., Ferris S., Sabbagh M., Gauthier S., Auer S., Shulman M.B., Abou-Saleh M.T., Katona C., Kumar A. (2011). Staging dementia. Principles and Practice of Geriatric Psychiatry.

[26] Kim H.K., Kim M., Kim S., Kim M., Chung J.H. (2004). Effects of green tea polyphenol on cognitive and acetylcholinesterase activities. Biosci. Biotechnol. Biochem..

[27] Lee B., Sur B., Kwon S., Yeom M., Shim I., Lee H., Hahm D.H. (2013). Chronic administration of catechin decreases depression and anxiety-like behaviors in a rat model using chronic corticosterone injections. Biomol. Ther. (Seoul).

[28] Juneja L.R., Chu D.C., Okubo T., Nagato Y., Yokogoshi H. (1999). l-Theanine—A unique amino acid of green tea and its relaxation effect in humans. Trends Food Sci. Technol..

[29] Yokogoshi H., Mochizuki M., Saitoh K. (1998). Theanine-induced reduction of brain serotonin concentration in rats. Biosci. Biotechnol. Biochem..

[30] Yokogoshi H., Kobayashi M., Mochizuki M., Terashima T. (1998). Effect of theanine, r-glutamylethylamide, on brain monoamines and striatal dopamine release in conscious rats. Neurochem. Res..

[31] Yamada T., Terashima T., Kawano S., Furuno R., Okubo T., Juneja L.R., Yokogoshi H. (2009). Theanine, gamma-glutamylethylamide, a unique amino acid in tea leaves, modulates neurotransmitter concentrations in the brain striatum interstitium in conscious rats. Amino Acids.

[32] Zwyghuizen-Doorenbos A., Roehrs T.A., Lipschutz L., Timms V., Roth T. (1990). Effects of caffeine on alertness. Psychopharmacology (Berl.).

[33] Schmidt A., Hammann F., Wölnerhanssen B., Meyer-Gerspach A.C., Drewe J., Beglinger C., Borgwardt S. (2014). Green tea extract enhances parieto-frontal connectivity during working memory processing. Psychopharmacology (Berl.).

[34] Feng L., Li J., Kua E.H., Lee T.S., Yap K.B., Rush A.J., Ng T.P. (2012). Association between tea consumption and depressive symptoms in older Chinese adults. J. Am. Geriatr. Soc..

[35] Niu K., Hozawa A., Kuriyama S., Ebihara S., Guo H., Nakaya N., Ohmori-Matsuda K., Takahashi H., Masamune Y., Asada M. (2009). Green tea consumption is associated with depressive symptoms in the elderly. Am. J. Clin. Nutr..

[36] Feng L., Yan Z., Sun B., Cai C., Jiang H., Kua E.H., Ng T.P., Qiu C. (2013). Tea consumption and depressive symptoms in older people in rural China. J. Am. Geriatr. Soc..

